# Nondestructive Imaging and Quantification of Composition in 2D MoS_2_ and V‑Doped MoS_2_ by the Auger Scatterplot Method

**DOI:** 10.1021/acs.jpcc.5c05299

**Published:** 2025-11-18

**Authors:** L’ubomír Vančo, Ravi K. Biroju, Mário Kotlár, Viliam Vretenár, Dipak Maity, Tharangattu N. Narayanan

**Affiliations:** 1 Centre for Nanodiagnostics of Materials, Faculty of Materials Science and Technology, Slovak University of Technology in Bratislava, Vazovova 5, Bratislava 812 43, Slovakia; 2 School of Advanced Sciences, Division of Physics, 385263 Vellore Institute of Technology, Chennai, Tamil Nadu 600127, India; § Materials & Interface Engineering Laboratory, 382966 Tata Institute of Fundamental Research, Sy No 36/P Serilingampally Mandal, Hyderabad 500046, India; ⊥ Centre for Nanoelectronics & VLSI Design, 385263 Vellore Institute of Technology, Chennai, Tamil Nadu 600127, India

## Abstract

Molybdenum disulfide (MoS_2_) monolayers have emerged as promising materials for a variety of applications. Their behavior depends critically on surface composition; therefore, careful characterization is necessary to describe their properties accurately. Although Auger electron spectroscopy (AES) is a standard method capable of addressing this issue, it suffers from beam-induced damage and variation of spectral features in complex samples. To overcome these limitations, we employed correlative analysis to examine MoS_2_ and V-doped MoS_2_ 2D surfaces by using Auger scatterplots. As we demonstrate, this method enables the nondestructive imaging and assessment of the lateral and depth distributions of the elements and provides a remarkably convenient way to estimate S-rich/depleted regions. The scatterplot technique indicates that V doping in MoS_2_ retards desulfurization in an Ar/H_2_ plasma environment. By reducing the electron dose, the analysis using scatterplots can improve the accuracy of AES by up to 30%. The Auger scatterplot method provides insight into the affinity or independence of surface constituents through quantitative relationships, enabling separate analysis of the characteristic areas within a complex sample. These findings are supported by Raman spectroscopy and transmission electron microscopy, which highlight the effectiveness of the Auger scatterplots and their potential for examining the surfaces of 2D materials. Auger maps also show a strong correlation with photoluminescence features in MoS_2_ monolayers, thereby illustrating the overlap with practical applications.

## Graphical abstract

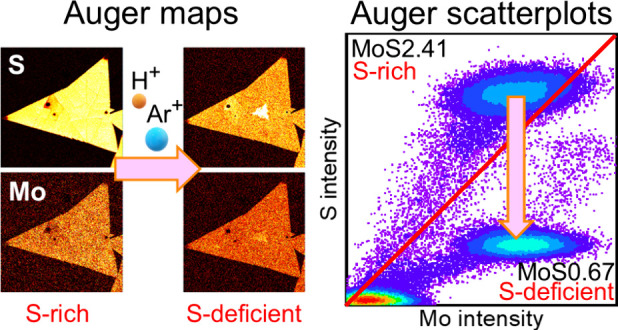

## 1 Introduction

Molybdenum disulfide (MS) monolayers are among the most extensively studied two-dimensional (2D) materials. Their potential applications in photodetectors, sensors, memory devices, photovoltaics, electrocatalysis, and biosensing are noteworthy. However, challenges persist regarding their stability, aging,^1^ serial integration, and susceptibility to contamination. Recently, V-doped MS (VMS) has also attracted significant attention due to its novel properties. By controlling the concentration of dopants, the electron structure can be tailored, and doping can bring magnetism and break the time-reversal symmetry in VMS, which could be beneficial for future valleytronics applications.^2,3^ However, it also generates defects in the MS lattice and exerts strain on the structure.

Doping, strain, defects, and surface modifications^4^ have substantial implications to the performance of MS/VMS-based devices. Surface modification is a critical factor because affiliation with external molecules, supporting substrates, the occurrence of charge transfer, or the presence of contaminants and inhomogeneities alters the properties of MS.^5^ Substituting S in the outermost atomic layer of MS for yttrium leads to ohmic contacts in Au/MS contact structures^6^ or atomically clean van der Waals (vdW) interfaces such as In/MS and Au/MoS_2(1–*x*)_Se_2*x*_, resulting in ohmic-like electron transport.^7,8^ The photoluminescence (PL) of monolayer MS can be modulated by adjacent layers,^9^ organic species,^10^ and by direct doping with substitutional atoms.^11^ Additionally, PL is affected by positioning on different substrates or by surface adsorbates originating from air/ambient, moisture, or residues from the growth process. Oxygen bonded to sulfur vacancies may enhance PL by a factor of 1000.^12^ Conversely, unpasivated S-vacancies give rise to new PL states.^13^ In hybrid MS/graphene photodetectors, the species adsorbed on the 2D surface can modulate the doping of the graphene layer from n-type to p-type, resulting in a change of the sign and magnitude of the photocurrent.^14,15^

The thickness of MS/VMS layers is influenced by many factors, including the type of substrate^16^ and contamination. The substrate affects the vdW gap in the substrate/layer architecture. For instance, the Au–MS system exhibits a vdW gap of ∼0.45 nm,^17^ while a more conventional SiO_2_–MS interface possesses a value of ∼0.475 nm (distance to the horizontal plane of Mo atoms).^18^ In contrast, it is evident that contamination species have a significant impact on the thickness. The most common type of contamination, adsorbed hydrocarbons,^19^ polymerizes under the electron beam in electron microscopes, leaving cross-linked carbon on the surface.^20^ Unreacted S species from the growth residing on top of layers also contribute to an increase in the thickness and roughness. Widely used polymer transfer or mechanical exfoliation has the potential to introduce polymer molecules into the vdW gap in between the transferred layers. This may result in thickness variations from the expected value, and a bilayer may reach 4 nm.^21^

This account of a limited number of examples demonstrates the demand for controllable, homogeneous, and clean surfaces, as well as for the methods for practical surface and interface analysis at the (sub)­nanometer scale, including unconventional techniques.^8,22^ Auger electron spectroscopy (AES) is a well-established technique for examining nanostructures. In the field of 2D materials, it sometimes complements methods such as Raman spectroscopy, X-ray photoelectron spectroscopy (XPS),^23^ or scanning transmission electron microscopy (STEM). The lateral range achievable with AES, spanning from micrometers to nanometers, is advantageous over the micrometer range achievable in XPS, which is too broad to describe smaller 2D crystals in detail. Conversely, STEM provides a too narrow field of view with the lateral resolution down to the atomic level, which does not allow for analysis in larger regions. In terms of depth resolution, AES is exceptional. In the case of MS on SiO_2_, the information depth reaches 1–1.5 nm, whereas for XPS, the value of ∼7–8 nm or more is typical. Together with a strong lateral resolution of ∼10 nm, this makes AES extremely sensitive to the outermost atoms in the studied layers, including contamination and adsorbates.

For the analysis of surfaces, AES typically employs point or area Auger spectra or Auger maps. However, the full potential of AES applied to 2D materials cannot be fully realized through Auger spectra alone, as they contain only average information related to the analyzed point or area. Therefore, Auger mapping appears to be a more robust method due to its sensitivity to spatial variations in surface composition and sample thickness. However, the conclusions based on maps may be and usually are only qualitative or tentative.

In this context, we demonstrate how the correlation of two different elemental Auger maps into an Auger scatterplot can significantly enhance the analysis of the MS and VMS 2D layers. The correlation is achieved by combining two Auger maps for two different elements into a two-dimensional *x*–*y* diagram (scatterplot) as two-dimensional histograms. The technique enables a reliable qualitative and quantitative inspection of surface chemistry. The rationale for the suggested approach is that except in cases involving highly homogeneous materials and atomically clean surfaces, Auger spectra acquired at different points/areas within a studied sample tend to be inconsistent. These discrepancies can be observed in the electron background level, variations in signal intensities, and differing intensities due to carbon, surface oxidation, and other species. As these effects accumulate in response to the excitation electron beam, the shape of the resulting electron spectra is altered. In secondary electron images, these variations may sometimes be visible, sometimes invisible, or even deceptive. If the spectra are recorded at distant points or areas, their interpretation may be confusing due to a lack of context and discontinuity in the choice of the region of interest. Furthermore, a high-quality Auger spectrum requires relatively high currents and long dwell times, which can cause damage to sensitive samples. Since 2D materials consist of atomically thin layers, they are highly susceptible to external stimuli, whether kinetic or chemical.

In this study, MS and VMS samples grown by chemical vapor deposition (CVD) were examined using Auger scatterplots in their as-grown and S-depleted states, the latter of which was induced by plasma treatment. The experiments were also supported by additional analytical techniques, including Raman spectroscopy and electron energy loss spectroscopy (EELS). Quantifying the Auger scatterplots allowed us to assess the lateral and vertical distribution of the surface elements, significantly improving our estimate of surface composition due to the reduced electron impact. Using the scatterplot method, we also found that the VMS layers are more resistant to the impact of fine downstream plasma than MS layers. These findings demonstrate the remarkable effectiveness of the Auger scatterplot method in characterizing MS/VMS surfaces and its potential for advanced surface analysis in the broader family of 2D materials. To show the impact of surface composition on the optical properties exploited in practical applications, we qualitatively correlate Auger maps with PL mapping for the first time. This illustrates variations in PL intensity, full width at half-maximum (fwhm), and energy in conjunction with the spatial distributions of surface constituents.

## 2 Experimental Section

### 2.1 Samples

To study the Auger scatterplot method, we used our own synthesized MS and VMS samples as well as reference MoS_2_ and SiO_2_ samples. The reference MoS_2_ monocrystal was purchased from hq graphene (Groningen, NL) as a standard with established stoichiometry to acquire the reference values for S and Mo intensities. Prior to the acquisition of the standard spectra, the surface of the crystal was exfoliated by Scotch tape, and the monocrystal was immediately inserted into a UHV chamber of the Auger microprobe (∼10^–7^ Pa). The exposed crystal face was then scanned for contamination-free areas in order to acquire the intensity references for S-KLL and Mo-MNN Auger peaks at 148 eV and 184–220 eV, respectively. The reference O-KLL peak (at 500 eV) of SiO_2_ was obtained using bare Si/SiO_2_ substrates, which also served as supports for the synthesized MS/VMS samples. The standard Auger spectra were used as a starting point for quantitative operations carried out on synthesized MS and VMS structures.

Grown MS samples subjected to the analysis were synthesized using a two-zone CVD method with a quartz tube (∼120 cm in length and ∼5 cm in inner diameter). MoO_3_ (4 mg) was used as the precursor placed in an alumina boat, with a Si/SiO_2_ (300 nm thick) substrate positioned on top. Sulfur (300 mg) was placed in another alumina boat, with the sulfur boat situated in zone I and the precursor boat situated in zone II. The sulfur zone was maintained at 210 °C, while the precursor zone was heated to 710 °C for 30 min. The growth process lasted ∼15 min, with nitrogen gas serving as a carrier at a flow rate of ∼187 sccm. The conditions for VMS growth were similar, except for 50 mg of V_2_O_5_, which was added to the MoO_3_ precursor with a small amount of KCl to reduce the metal decomposition temperature. Further details regarding the synthesis can be found in our previous works^24,25^ and are also shown in the Supporting Information, Figure S1.

### 2.2 Plasma Treatment of MS and VMS Samples

To modify the S content in the synthesized MS and VMS samples, the 2D layers were etched in low-energy downstream plasma with Ar–H_2_ (95%–5%) working gas. The analyzing and etching cycles were performed in an intermittent manner without exposing the samples to the atmosphere. During the etching process, the samples were stored in a load lock chamber that was directly connected to the Auger microprobe. The load lock was equipped with a plasma generator GV10x P6 Downstream Asher from ibss Group, Inc., and the samples were exposed to 50 W plasma at a pressure of 0.1 Pa. The first etching cycle lasted 30 s, and this was followed by two additional cycles of 10 s each, resulting in a total etching time of 50 s. The distance between the samples and the plasma source was 250 mm.

### 2.3 Auger Analysis

Secondary electron imaging and Auger analyses (spectra and maps) were carried out on a JEOL JAMP-9510F Auger microprobe equipped with a field emission Schottky electron gun and hemispherical electron analyzer. All samples were excited by 10 keV electrons at 10 nA current, which was calibrated by a Faraday cup. The incident and emission angles were set to 30°, and the constant retard ratio regime was employed with 0.6% energy resolution.

Auger spectra were captured in the direct form EN­(E) with a dwell time of 100 ms. Given that Auger intensity is proportional to the primary beam current and dwell time, the spectra were normalized to 1 nA × 1 ms to extract normalized Auger intensities for the S-KLL, Mo-MNN, and O-KLL Auger transitions. In the case of VMS samples, we occasionally used differentiated Auger spectra d­(EN­(E))/dE followed by 11-point Savitzky–Golay smoothing to access less intense vanadium peaks as shown in the Supporting Information.

The Auger maps were acquired with 1 ms dwell time for S and O, and with 3 ms dwell time for C and Mo. With the exception of the number of acquisitions, which varied from 1 to 3 depending on the signal-to-noise ratio, the measurement conditions remained constant. The signal intensities for each pixel in the Auger maps were calculated at P (peak) and B (background) positions as P–B. The positions for P and B were determined prior to the mapping, separately for each chemical element, and are shown in the Supporting Information. The Auger intensities at each pixel extracted from the Auger maps were also normalized to 1 ms × 1 nA. Prior to the evaluation in the scatterplots, the data were smoothed by a linear box filter in the internal software Auger Master (JEOL).

### 2.4 Complementary Analysis

Raman measurements with Ar 514 nm excitation were carried out on a Raman system by Spectroscopy & Imaging GmbH, Germany. The system was equipped with a Peltier-cooled detector, a confocal Raman microscope Olympus BX51, and a 100× objective lens (numerical aperture = 0.9) in the backscattering configuration. The power employed was <2 mW, and the grating was 600 gr/mm.

Photoluminescence mapping was investigated by using a Witec Alpha 300R system with 532 nm excitation. A confocal microscope with a 50× objective (NA = 0.8), a diffraction grating (150 gr/mm), and a Peltier-cooled CCD camera was employed.

For scanning transmission electron microscopy analysis (STEM), the MS layers were transferred by polymer assistance to a TEM grid. The samples were characterized with a JEOL JEM ARM200CF equipped with image and probe correctors and operated at 80 kV. The images were recorded in STEM mode by an annular dark field (ADF) detector, and the chemical composition of the layers was investigated by electron energy loss spectroscopy (EELS) using a GIF Quantum 965 ER Gatan Imaging Filter with Dual EELS capability.

## 3 Results and Discussion

To study the behavior of Auger scatterplots in 2D layers, two types of samples were prepared by CVD, namely, MoS_2_ and V-doped MoS_2_ on Si/SiO_2_ substrates. For the sake of brevity, they are herein termed MS and VMS, respectively.

First, Raman spectroscopy was used to verify the number of layers in the prepared 2D crystals. Figure S2 shows the Raman spectra acquired on four different 2D MS crystals. The Raman spectra exhibit two features typical of MoS_2_ 2D material, an in-plane E_2g_ mode at 384.7 cm^–1^, and an out-of-plane A_1g_ mode at 404 cm^–1^ (Table S1). In a thicker layer, the two modes shift to 372.5 cm^–1^ and 408.5 cm^–1^, respectively. The separation Δ between E_2g_ and A_1g_ modes in the thin areas proves the presence of a monolayer (Δ = 19.3 cm^–1^), while the bulkier area with Δ = 24 cm^–1^ is equivalent to 3 monolayers.

Second, the presence of V in VMS layers was verified by AES, Figure S3, through the observation of a V-LMM Auger peak at 432 eV in differentiated Auger spectra. While V is present in the layers, it is not distributed over the SiO_2_ substrate in the vicinity of the 2D layers. The amount of V in the layers is estimated to ∼2% based on the intensity of the V-LMM peak.

### 3.1 Rationale for the Auger Scatterplot Method

To emphasize the motivation for using Auger scatterplots, we first carried out the classical AES analysis. We also used these results as a reference point to compare the effectiveness of standard AES analysis and the Auger scatterplot method. In Figure 1a, secondary electron images for three distinct MS layers and one VMS layer are presented. White rectangles denote the areas for the acquisition of Auger spectra shown in Figure 1b—MS sample—and Figure 1c—VMS sample. The MS spectra exhibit variations in S and Mo signals and in background level due to the varying amount of contamination on the surface of the layers evident from the presence of a C-KLL peak at 265 eV. In the VMS layer, the S and Mo intensity modulation can be attributed to the variations in the layer thickness. The thickness disparities throughout the VMS grain can be identified from variation in the O-KLL peak associated with the SiO_2_ substrate. The thickness *t* can be evaluated based on the exponential decrease of the O-KLL signal coming from the substrate oxide, which is covered by an overlayer;^26–28^ in our case, the overlayer is the 2D crystal:

$$t=\lambda_{O}^{MS}\cos\theta \ln\left(I_{O}^{\infty }/I_{O}\right)$$ 1

Here, λ_*O*_^*MS*^ is the effective attenuation length (EAL) of the O-KLL Auger electrons (∼500 eV) in the MS material, θ is the emission angle, and *I*_*O*_^∞^ and *I*_*O*_ are the measured O-KLL intensities at the clean oxide substrate and at the 2D layer, respectively. To calculate *t*, we prefer the O-KLL peak to the Si-LVV peak, as the latter is more suitable for thinner structures with thicknesses up to ∼1 nm.^29^ The value of EAL is accessible via the NIST SRD 82 database,^30^ see Figure S4, or can be estimated experimentally,^31^ *I*_*O*_^∞^ = 21.3 cts/msec × nA is taken from the reference SiO_2_ spectrum in Figure S5a. For the surface composition, the coefficient *x* in a monolayer MoS_*x*_ can be estimated as

$$x=2\frac{I_{S}}{I_{Mo}}\frac{I_{Mo}^{1}}{I_{S}^{1}}$$ 2

Here, *I*_*S*_ and *I*_*Mo*_ are the measured Auger intensities for the Mo-MNN and S-LVV Auger transitions in a sample under study and *I*_*Mo*_^1^ = 2.65 cts/msec × nA and *I*_*S*_^1^ = 27.2 cts/msec × nA are reference intensities for sulfur and molybdenum in the MS monolayer. These values are derived from the bulk MoS_2_ reference crystal, see Figure S5b, after considering EAL and backscattering contributions,^32^ as outlined in the Supporting Information.

**Figure 1:**
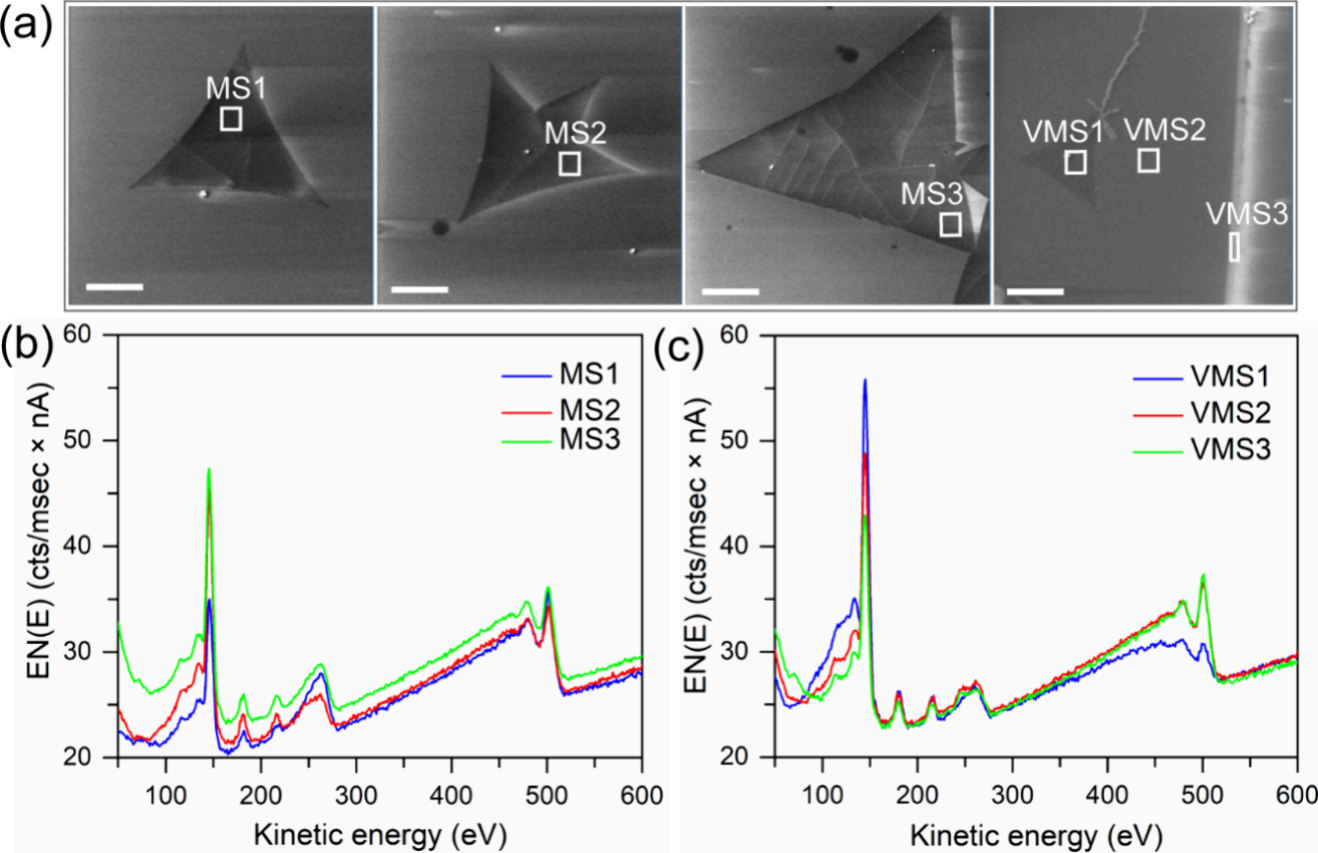
(a) SEM images of three different MS layers and one VMS layer, (b) Auger electron spectra from the MS layers marked MS1 to MS3 in the SEM images, and (c) Auger electron spectra from the VMS layer acquired on the areas marked VMS1 to VMS3 in the corresponding SEM image. The scale bar in all the figures is 20 μm.

Equations 1 and 2 applied to the layers in Figure 1a provide the values summarized in Table 1. The thicknesses, with the exception of VMS1, correspond to the monolayers plus some contamination, coming from carbon and/or extra sulfur unreacted in the growth process. However, according to the calculated thickness, VMS1 is a contaminated bilayer.

**Table 1:** Calculated Thicknesses and Stoichiometries for the Layers Shown in Figure 1

parameter	MS1	MS2	MS3	VMS1	VMS2	VMS3
thickness (nm)	0.67	0.83	0.78	1.62	0.72	0.63
*x* in MoS_*x*_	1.78	1.86	1.99	1.81	1.69	1.61

It is seen that the calculated stoichiometries are underestimated, since *x* < 2. The cause is the susceptibility of extremely thin MS/VMS layers to electron beam damage. Additionally, there is also a lack of complete information regarding the lateral and depth homogeneity of the layers. Consequently, a more robust method for examining MS/VMS layers is required.

To resolve these issues, correlative Auger mapping is suggested to elucidate the relationship among the constituents in 2D layers. The correlation can be achieved via scatterplots—also known as 2D histograms—generated from the collected Auger maps of the examined elements such as S, Mo, C, and O.

### 3.2 Construction of Auger Scatterplots

The construction of an Auger scatterplot for S and O is illustrated in Figure 2. First, the Auger maps of S and O are acquired at the same resolution and size and with the same position of the MS layers as shown in Figure 2a. The maps illustrate a group of few-layered MS crystals arranged in a star-like shape. The analysis of such a structure is interesting due to continuous modulation of S and O intensities resulting from variations in the thickness of the deposit.

**Figure 2:**
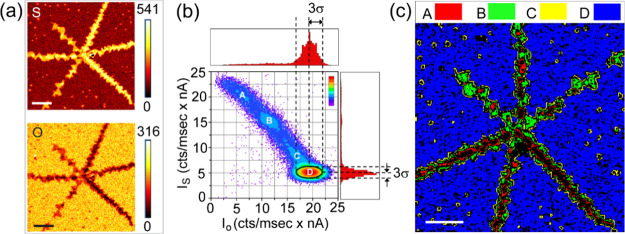
(a) S and O Auger maps of grouped MS layers, (b) corresponding Auger scatterplot of S and O intensities derived from the S and O maps shown together with their intensity distributions, and (c) phase map for clusters A–D shown in the previous scatterplot. The scale bar in all the figures is 10 μm.

Second, a linear box filter is applied to both maps to reduce the statistical variance of the intensity distributions in S and O signals, but with no effect to their mean values as documented in Figure S6. Finally, each pixel in the S map is correlated with the corresponding pixel in the O map by extracting the Auger intensities collected at that pixel. The pixel is then represented in the scatterplot as one point with the corresponding values of S and O intensities (*I*_*S*_ and *I*_*O*_) graphed using vertical (*I*_*S*_) and horizontal (*I*_*O*_) axes, respectively. As illustrated in Figure 2b, the S–O scatterplot is composed of all of the pixels contained within the maps. All maps presented in this study are 256 × 256 pixels, resulting in 65,536 points in the scatterplots. This provides a rich statistical set, enabling a reliable determination of mean values despite noisy signals.

The density of the scatter points in the scatterplot is color-coded (violet = low, red = high), allowing the identification of the densest clusters here denoted as A, B, C, and D. Since the density of the data in the clusters is higher than in the rest of the scatterplot, they represent the most frequent areas in the maps. Adjacent to the scatterplot, the histograms of *I*_*S*_ and *I*_*O*_ values are displayed to emphasize the correspondence with the plotted data and highlight their statistical nature. The size of the clusters and their centers determine 3σ values and mean values of the respective Gauss distributions in the filtered normalized *I*_*S*_ and *I*_*O*_. In this study, we have used the mean values to quantify the composition of the samples despite rather high statistical variance. The statistical variance can be reduced by higher excitation current, higher dwell times, or higher number of sweeps during the measurement. As all three procedures enhance the electron dose imposed on the sample, they appear to be a compromising solution between signal-to-noise ratio and beam-induced damage.

Clusters A, B, C, and D seen in the scatterplot are situated on a continuous background of evenly distributed, low-density data described by a concave diagram. As we demonstrate in the following paragraph, the concave diagram corresponds to an S-on-O structure, that is, an O-containing substrate covered with MS layers on top. Conversely, a convex diagram would represent the other case of the O-on-S deposit. Therefore, it can be concluded that O is lacking on the top of the MS structure. This phenomenon can be attributed to the presence of additional S deposits on the surface coming from S remnants not fully consumed during the growth process, now preventing O contamination. The presence of the extra S is contradicted by Table 1, where the S content does not reach two S atoms per one Mo atom. However, it will be demonstrated that the layers are indeed sulfurized and that the values in the table are underestimated due to beam damage.

The data points contained in the scatterplot may be arbitrarily selected point-by-point and represented in a phase map, as shown in Figure 2c. This method allows the individual Auger maps to be synthesized into a unique image facilitating the visualization of specific two-element correlations. As indicated by the phase map, clusters A, B, C, and D represent areas of varied thickness of the S deposit (A = high, D = low). Notably, cluster D represents a continuous S-containing coverage on the oxide surface.

### 3.3 Layer Order from Auger Scatterplots

The existing theoretical models can be used to quantitatively explain the interpretation of the concavity and convexity of the diagrams along with the layering. Qualitatively, Figure 3a shows two S and C Auger maps of the MS sample alongside the corresponding S–C scatterplot in Figure 3b.

**Figure 3:**
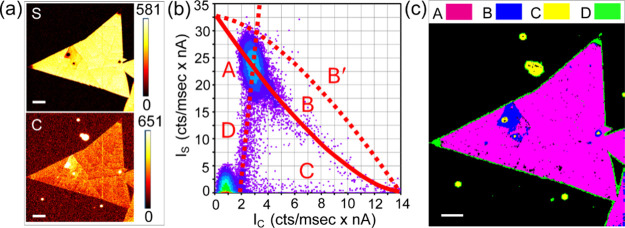
(a) S and C Auger maps in the MS monolayer, (b) corresponding Auger scatterplot, and (c) phase map constructed from the main clusters A–D seen in the scatterplot. The scale bar in all the figures is 20 μm.

The scatterplot contains a dominant cluster A, data points along curve B, data points along the *x*-axis (C), and data points along line D. The interpretation of the regions A–D is clearly outlined in a phase map in Figure 3c. Region A corresponds to the dominant area of the 2D layer with a little carbon deposit; region B is a part of the layer with enhanced contamination; region C is bulky contamination on the substrate and the top of the crystal; finally, region D represents the edges of the layer.

When considered quantitatively, Auger intensity *I*_*X*_ for a thin overlayer with thickness *t* made of element X is expressed by^26^

$$I_{X}=I_{X}^{\infty }\left\{1-\exp\left[-t/\left(\lambda_{X}^{X}\cos\theta \right)\right]\right\}$$ 3a

where *I*_*X*_^∞^ is the Auger signal from bulk *X*, θ is the emission angle, and λ_*X*_^*X*^ is the EAL of the Auger electrons originating from element *X* and spreading through layer *X*. On the other hand, the Auger signal *I*_*Y*_ of element *Y* in a layer buried under the layer *X* is given by^26^

$$I_{Y}=I_{Y}^{\infty }\exp\left[-t/\left(\lambda_{Y}^{X}\cos\theta \right)\right]$$ 3b

where *I*_*Y*_^∞^ is the Auger signal from uncovered layer *Y* and λ_*Y*_^*X*^ is the EAL of the Auger electrons from element *Y* spreading through layer *X*. In the case when the top layer X = C and the underlayer Y = S, that is C-on-S, we can combine eqs 3a and 3b through *t* to obtain a relationship between the intensities *I*_*S*_ and *I*_*C*_ measured in the Auger maps:

$$I_{S}=I_{S}^{\infty }{\left(1-\frac{I_{C}}{I_{C}^{\infty }}\right)}^{\lambda_{C}^{C}/\lambda_{S}^{C}}$$ 4a

where *I*_*C*_^∞^ can be approximated by the maximum value of *I*_*C*_ given by the bulk clusters of C contamination. In the other case when X = S and Y = C, that is, S-on-C, we obtain a different relation:

$$I_{S}=I_{S}^{\infty }\left[1-{\left(\frac{I_{C}}{I_{C}^{\infty }}\right)}^{\lambda_{C}^{S}/\lambda_{S}^{S}}\right]$$ 4b

In Figure 3b, curve B represents eq 4a, and curve B′ represents eq 4b. We can therefore conclude that the examined MS monolayer contains C-on-S, since the scatterplot follows curve B. In the enumeration, we used a practical relation for the exponents in eqs 4a and 4b: λ_*X*_/λ_*Y*_ ≈ (*E*_*X*_/*E*_*Y*_)^0.75^, where *E* is the energy of Auger electrons for elements *X* and *Y*. This equation provides an approximate value for λ_*C*_^*C*^/λ_*S*_^*C*^ ≈ λ_*C*_^*S*^/λ_*S*_^*S*^ ≈ 1.53, which is used to plot curves B and B′.

### 3.4 Surface Composition from Auger Scatterplots

The other aspect that can be conveniently approached with Auger scatterplots is the estimation of Mo/S composition and S enrichment/depletion of the surfaces. For that purpose, an S–Mo scatterplot can be constructed from S and Mo Auger maps as shown in Figure 4a. As is apparent from the maps, the inspected layer is seemingly homogeneous in terms of S and Mo intensities, but there is a local variation due to the enhanced contamination.

**Figure 4:**
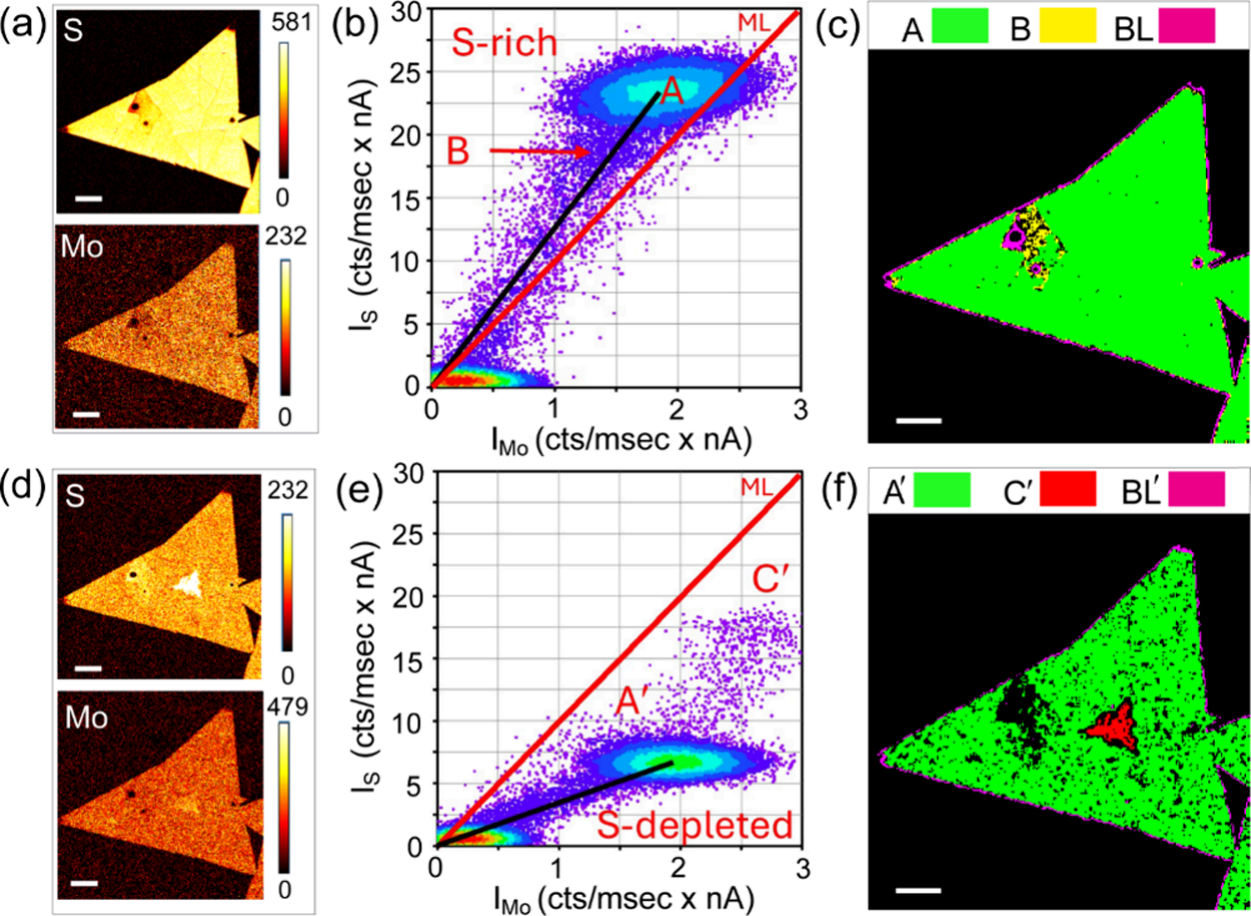
(a) S and Mo Auger maps in the as-grown MS layer, prior to plasma treatment, (b) corresponding Auger scatterplot with a red line dividing the diagram into halves to summarize the S-rich and S-depleted surface, (c) phase map to clusters A and B and along the black line shown in the scatterplot, and (d–f) the analogues for the same MS layer subjected to Ar/H_2_ plasma for 30 s, showing S-depletion after the plasma treatment. The scale bar in all the figures is 20 μm.

The corresponding S–Mo scatterplot in Figure 4b contains clusters A and B as markers for the layer and the local defect with higher C contamination, as suggested in the phase map in Figure 4c. The points along the black line (BL) can be again interpreted as the edges of the crystal.

The ideal stoichiometry in a monolayer is shown as a red line in the scatterplot, denoted as ML. It runs through the points where *x* = 2 in MoS_*x*_ and allows the assessment of S enrichment/depletion for clusters A and B. As the A and B clusters (the black line) are situated above the reference line, they indicate an oversulfurized surface. Based on the mean intensities represented by the centers in the clusters A and B in the S–Mo scatterplot, the stoichiometry according to eq 2 is MoS_2.41_ indicating extra S on the surface. This finding is in line with STEM analysis (Figure S7), which also shows S remnants present on the surface. However, this observation contradicts Table 1, which identifies spot MS3 located at the same grain as MoS_1.99_. Therefore, the relative error in the stoichiometry calculated from the MS3 Auger spectrum is (2.41 – 1.99)/2.4 = 17.4% but can reach 26% in MS1 and even 30% in VMS3.

During the acquisition of the maps, the dwell time per pixel^33^ was 1–3 ms, whereas the dwell time for the Auger spectra in Figure 1 was 100 ms. From the calculated stoichiometries, we must conclude that the dose in MS3 is excessive and has a detrimental effect on the surface. Therefore, Auger scatterplots are more suitable for sensitive surfaces than point analysis. Conversely, the shorter dwell times result in an increased statistical variance due to a low signal-to-noise ratio. However, as follows from Figure S6, statistical filtering reduces the problem without impacting the mean values used for estimating the surface composition.

An accurate assessment of S content in MS/VMS layers may be important in the applications where S-deficient and S-vacant surfaces are desired, for example, in catalysis or electrochemistry. To simulate the impact of S-depletion on the scatterplots, the MS sample was exposed to Ar/H_2_ plasma for 30 s to preferentially sputter S sites and thereby initiate S-depleted surfaces. The results can be seen in Figure 4d–f showing Auger maps, a scatterplot, and a phase map for the S-depleted MS layer. The dominant cluster A′ representing the 2D crystal surface is now located under the reference line with the composition MoS_0.67_. In contrast to its previous location A above the reference line in the as-grown sample, which represents the S-rich surfaces, the location of the cluster under the reference line represents an S-depleted surface. The locally contaminated area B in the as-grown layer does not have its counterpart after plasma polishing in Figure 4e because the plasma removes most of the carbon deposits. Conversely, a new cluster C′ appears, representing a previously unseen thick MS center.

According to eq 3a, the first MS monolayer contributes ∼81% of the S signal, with an additional ∼14% contributed by the second layer and only ∼5% by the third layer. Therefore, in 3 layers of MS, the S signal is saturated, making it difficult to distinguish more than 3 monolayers of MS from bulk MS. Additionally, if oversulfurization on the surface of a monolayer adds 20% to the S signal (MoS_2.41_), it can be challenging to identify even a bilayer in MS/VMS. However, as the structure becomes S vacant, bulky cores are exposed as in the case of cluster C′. The effect of varying S content in the scatterplots was also investigated in VMS samples by applying Ar/H_2_ plasma in three steps, with total times of 30, 40, and 50 s. Figure 5a,b shows the Auger maps and a corresponding scatterplot from an as-grown VMS sample, while Figure 5c,d shows the same sample after the third step of plasma treatment, that is, after 50 s of etching. As evidenced by the Auger maps prior and after the treatment, the VMS sample consists of two monolayer crystals, with a thicker part located in the corner of the left-hand-side crystal. The scatterplot in Figure 5b indicates oversulfurization in the VMS surface (MoS_2.36_), and after 50 s of plasma etching, the monolayer part of the sample becomes S-depleted with the composition MoS_0.47_ (full black line). The thicker part is revealed after the plasma treatment, and S-depletion in this part is less pronounced than in the rest of the crystals (dashed black line). The shift of the dominant cluster from the upper part of the reference line to its lower part effectively indicates a transition from the S-rich to S-depleted surface. This result is supported by Raman spectroscopy, which evidence the amorphization in monolayers. However, the structural damage is less severe in the thicker layer (Figure S8).

**Figure 5:**
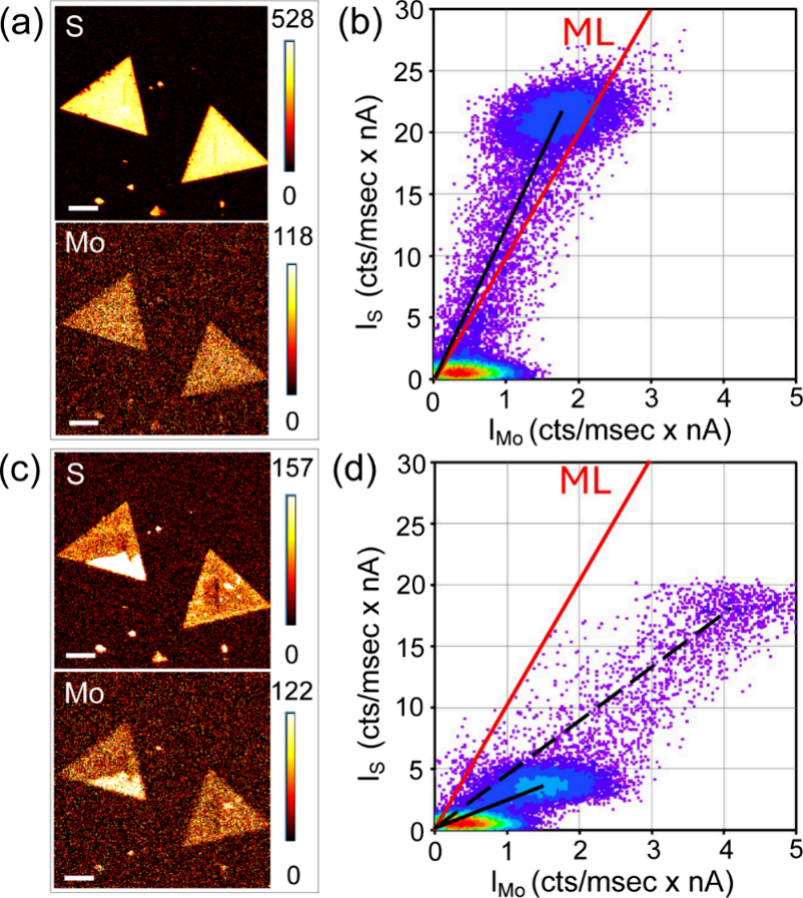
(a) S and Mo Auger maps in two as-grown VMS monolayer crystals prior to plasma treatment, (b) corresponding Auger scatterplot showing S-rich surface, (c) S and Mo Auger maps in the same sample after 50 s in the plasma, and (d) corresponding Auger scatterplot showing S-depletion in two regions: monolayer plus bulk part in the left crystal. The scale bar in all the figures is 5 μm.

Figure 6 presents a comparison of the stoichiometries calculated using eq 2 in the VMS sample before and after plasma treatment. The monolayers and the thicker corner exhibit different responses to the plasma, with thicker layers demonstrating a lower susceptibility to S depletion. The stoichiometry coefficients are calculated from either Auger spectra or scatterplots. The two coefficients are highly consistent, except for the as-grown sample at 0 s, where they exhibit a significant deviation (V-MoS_2.36_ vs V-MoS_1.98_, 16% relative). As was discussed previously, the instability of S remnants under the electron beam in the MS samples is also applicable to the VMS. It leads to an underestimation of S content in point/area Auger spectra. Once the surface S adatoms have been removed by plasma, the differences in stoichiometry obtained from the spectra versus the maps tend to diminish. By comparing the S content in the MS and VMS layers after 30 s in the plasma (28% in MS, MoS_2.41_ to MoS_0.67_, and 38% in VMS, V-MoS_2.36_ to V-MoS_0.89_) we found that desulfurization was more pronounced in the MS material. A plausible explanation of the retardation effect in the VMS layers is the alloying of V within the MS structure. Since S is highly sensitive to external kinetic or chemical stimuli, even a small amount of V sites (∼2%) in the structure can enhance its resistance to plasma species.

**Figure 6:**
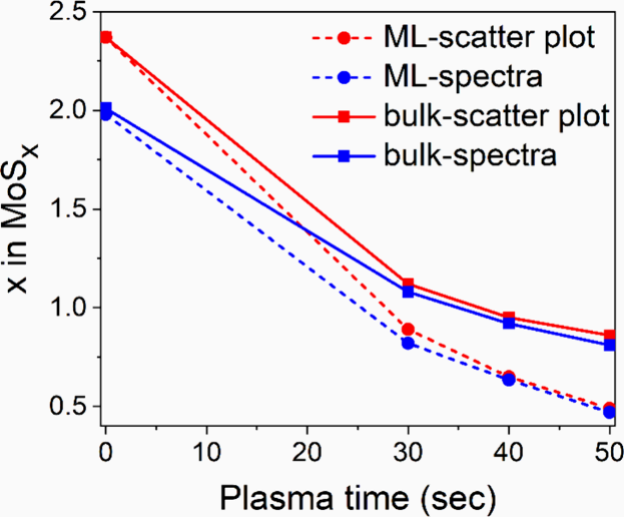
Stoichiometries obtained from either Auger scatterplots or Auger spectra in monolayers and bulkier VMS layers.

To demonstrate the impact of the reported spatial variations in surface elements on optical properties – including practical applications such as photoluminescence – we conducted AES and PL measurements on an individual MS crystal (Figure 7). A detailed study is required for a quantitative evaluation of the AES–PL correlation, which is beyond the scope of this work. However, we offer at least a preliminary qualitative assessment here.

**Figure 7:**
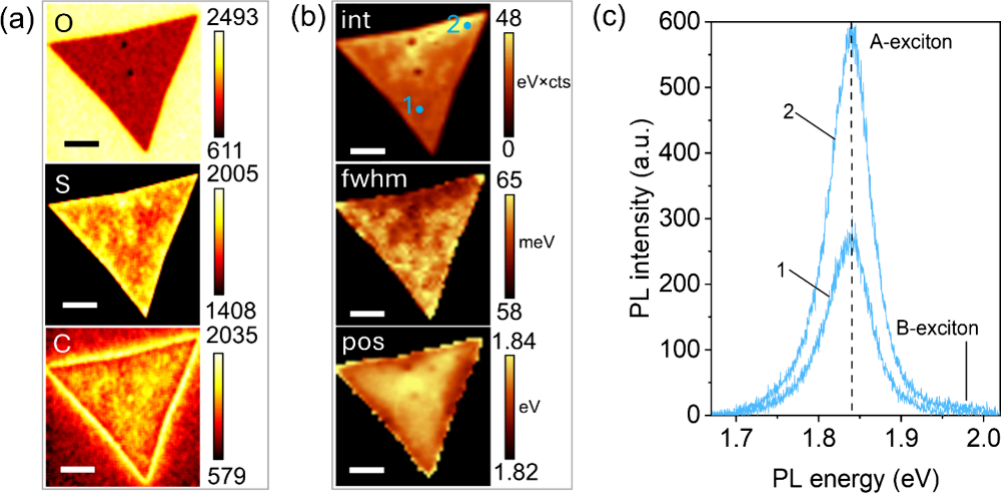
Correlative Auger and PL mapping. (a) Auger maps of an MS monolayer, (b) PL maps generated for the A exciton peak intensity, width, and position, and (c) two PL spectra acquired at points 1 and 2 shown in the PL intensity map. The scale bar in the figures is 10 μm.

Figure 7a shows the Auger maps of the surface distributions of O, S, and C, and Figure 7b shows the PL maps of the same grain. The PL maps illustrate the spatial distribution of PL peak position, intensity, and fwhm, and Figure 7c displays two PL spectra extracted from points 1 and 2, as indicated on the PL intensity map. As seen in Figure 7c, the PL spectra exhibit typical A-exciton maxima (∼1.84 eV) together with a broad B-exciton peak (∼1.96 eV). As the location of the grain varies, the peak position, intensity, and fwhm of the A-exciton peak are modified by locally dependent sulfurization and/or C contamination.

The Auger O map clearly shows two point defects of grainy coagulates, which have a lower O intensity due to their bulky nature. The rest of the map is nonuniform, but the intensity variations are too small to be measured accurately. In the S map, the point defects appear S-rich and are connected via a sulfur bridge. The surface of the grain at the edges and corners is S-rich, whereas the central part, which has three lobes, is S-depleted. The Auger C map is inversely related to the S map with higher C contamination in S-depleted regions. The interconnection of the Auger and PL maps is not straightforward and requires statistical analysis. However, the main areas of correlation are already obvious. First, the two grain-like defects shown in the O map tend to quench PL; conversely, the area between them, connected via the S bridge, behaves as a PL booster with a blueshift of about 10–20 meV. At the same time, PL intensity is inversely correlated with the PL peak width. Second, S-rich edge regions with lower surface carbon exhibit a redshift in the PL spectra. Conversely, whether directly or indirectly, carbon contamination causes a PL blueshift.

What remains unclear is the connection between the higher PL width in the corners of the grain and the higher PL intensity at the upper edge, which is not reflected in the Auger maps. This may be perhaps explained by additional optical measurements, such as correlative Raman–PL maps, as has recently been demonstrated in WS_2_ monolayers.^34^ The redshift of PL energy, the decrease in PL intensity, and the increase in PL peak width were assigned to higher doping and reduced strain, while the blueshift of PL energy was assigned to defects and reduced tensile strain. In our AES–PL maps, an increase in C contamination is connected with a blue-shift, while an increase in S content is more complex, sometimes correlating with a red-shift, sometimes with a blue-shift, and sometimes with an enhancement in intensity. The indicated complexity of the AES–PL correlation is interesting for further research to elucidate the detailed relationship between the surface composition and optical properties of MS layers.

Recently, a faster and more effective Auger mapping has been introduced through various technical solutions involved in the signal collection.^35^ The method helps to reduce the noise in Auger maps and the time needed for data acquisition. This should result in less damage being caused to the samples by the electron beam and lower statistical variance. The combination of the new technique with the Auger scatterplots method may be a valuable extension with the potential to generate significant results in the advanced analysis of 2D materials.

## 4 Conclusions

Despite the excellent lateral and depth resolution of Auger electron spectroscopy, the conventional evaluation of Auger spectra does not accurately describe the surfaces of CVD-grown MoS_2_ and V-MoS_2_ layers due to variations in surface composition and damage caused by the excitation electron beam. We have demonstrated that a quantitative evaluation of Auger scatterplots, correlating two Auger maps, removes these problems and yields strong, conclusive information about the lateral distribution of chemical elements, their vertical layering, and the accurate composition of their surfaces. Using the Auger scatterplot method, we have also achieved an improvement in stoichiometry estimation of up to 30% due to the reduced electron dose applied to the samples. A novel finding of the scatterplots is the different behavior of MoS_2_ and V-MoS_2_ 2D layers when subjected to soft downstream plasma, which is used for S depletion of the surfaces. Low vanadium doping concentrations (∼2%) decrease desulfurization in V-MoS_2_ compared to MoS_2_, likely due to the enhanced resistance of V-sites to plasma species compared to the extreme sensitivity of S-sites. Unlike the impact of plasma, the effect of the impinging electron beam is similar in both types of structure. Further work is needed to clarify the role of Auger scatterplots in the surface analysis of various other 2D materials, such as S-containing vertical or lateral heterostructures. Preliminary correlative Auger–PL mapping in the MoS_2_ monolayer indicates that the presence of C contaminants blueshifts the PL energy by ≤20 meV, while the presence of S remnants enhances the PL intensity and decreases the PL peak width by ≤7 meV. Our results justify the use of the Auger scatterplot method as a promising technique for the advanced, nondestructive analysis of MoS_2_ and V-doped MoS_2_ surfaces and interfaces, potentially expanding the range of analytical tools available in the field of 2D materials.

## Supplementary Material



## Notes

Schematic of the experimental setup for CVD growth of the samples, Raman spectra of the samples, Auger electron spectra from the V-doped samples, effective attenuation lengths in MoS_2_, reference Auger spectra, discussion of the attenuation and backscattering effects, filtering of Auger maps, STEM and EELS maps in MoS_2_, Auger maps and Raman spectra of plasma treated samples (PDF)
