# Supplementary material for: Nondestructive Imaging and Quantification of Composition in 2D MoS2 and V‑Doped MoS2 by the Auger Scatterplot Method
Source: J Phys Chem C Nanomater Interfaces. 2025 Nov 18;129(47):20995–1004. doi: 10.1021/acs.jpcc.5c05299 (PMC12670771; doi:10.1021/acs.jpcc.5c05299)
Supplement: Supplementary file 1 [file jp5c05299_si_001.pdf]

SUPPORTING INFORMATION

to

Non-destructive Imaging and Quantification of Composition in 2D MoS_2 and V-doped MoS_2 by the Auger Scatterplot Method

Ľubomír Vančo[1#], Ravi K. Biroju[1,2#], Mário Kotlár[1], Viliam Vretenár[1], Dipak Maity[3], Tharangattu N. Narayanan[3]

[1]Centre for Nanodiagnostics of Materials, Faculty of Materials Science and Technology, Slovak University of Technology in Bratislava, Vazovova 5, 812 43 Bratislava, Slovakia

[2] School of Advanced Sciences-Division of Physics, Vellore Institute of Technology Chennai-600127, Tamil Nadu, India

[3]Materials & Interface Engineering Laboratory, Tata Institute of Fundamental Research, Sy No 36/P Serilingampally Mandal, Hyderabad, 500046 India

ravi.biroju@stuba.sk; ravikumar.biroju@vit.ac.in
lubomir.vanco@stuba.sk
[#] equally contributing author

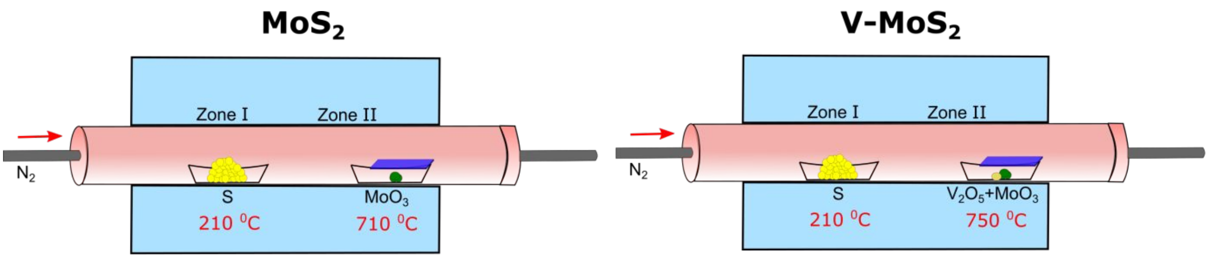

Figure S1. Schematic of the experimental setup used for CVD growth of MoS_2 and V-MoS_2 samples.

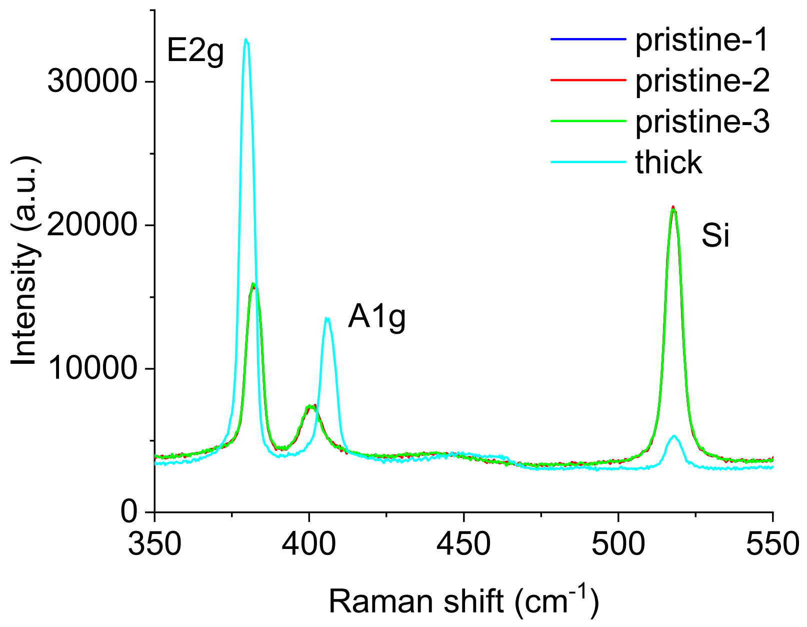

Figure S2. Raman spectra recorded from three different pristine 2D MS monolayers and from a thick area of the deposit.

Table S1. Frequencies of the E2g and A1g modes and their difference Δ showing 1ML of MoS_2 in the pristine crystals and ~3ML in the thick area.

	**A1g**	**E2g**	**Δ = A1g −E2g**
**1ML**	404 cm^{-1}	384.7 cm^{-1}	19.3 cm^{-1}
**THICK**	408.5 cm^{-1}	372.5 cm^{-1}	24 cm^{-1}

(a)

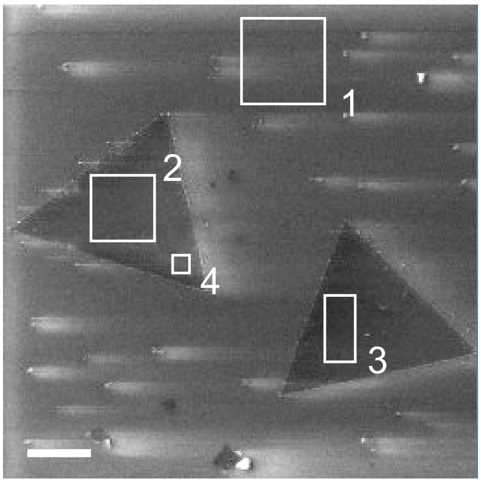

(b)

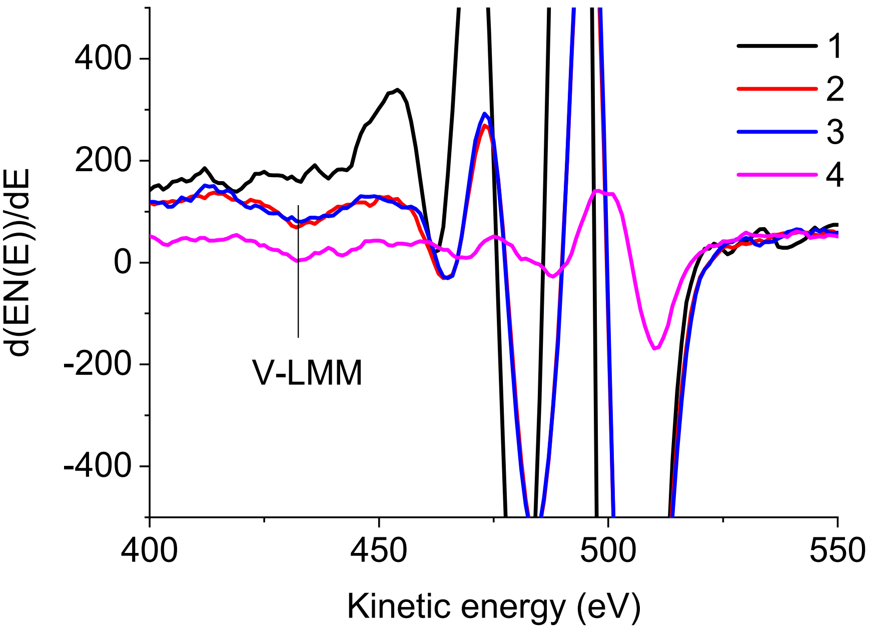

Figure S3. (a) SEI micrograph of a VMS sample with the areas analyzed by AES to confirm the presence of V. (b) detail of differentiated Auger electron spectra with V-LMM transitions present in the layer (areas 2, 3, 4) but not on the substrate (area 1). The scalebar is 4μm.

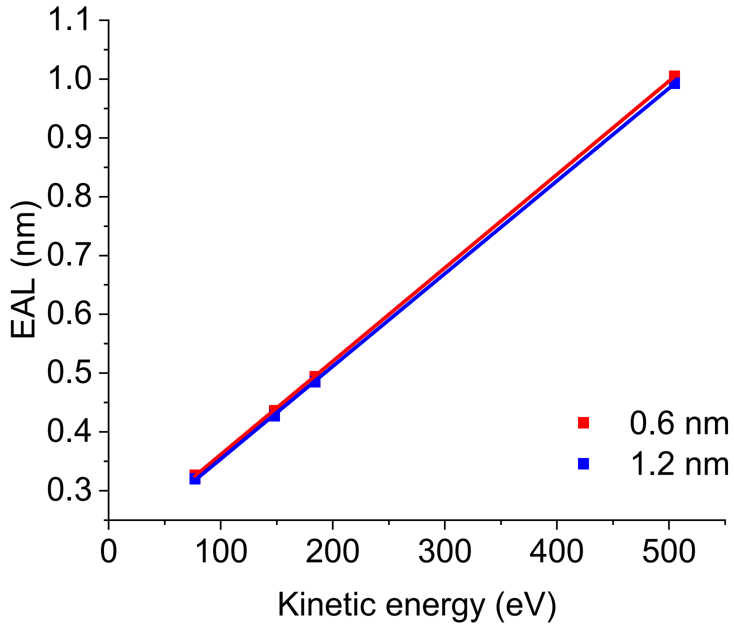

Figure S4. Average effective attenuation lengths in MoS_2 with two different thicknesses corresponding to ~1 ML and ~2MLs, calculated in the NIST SRD82 database − based on the TPP−2M estimation of inelastic mean free path. The points are calculated values for 75, 148, 184 and 505 eV electrons originating from Si, S, Mo and O elements, consecutively. The lines are linear fits.

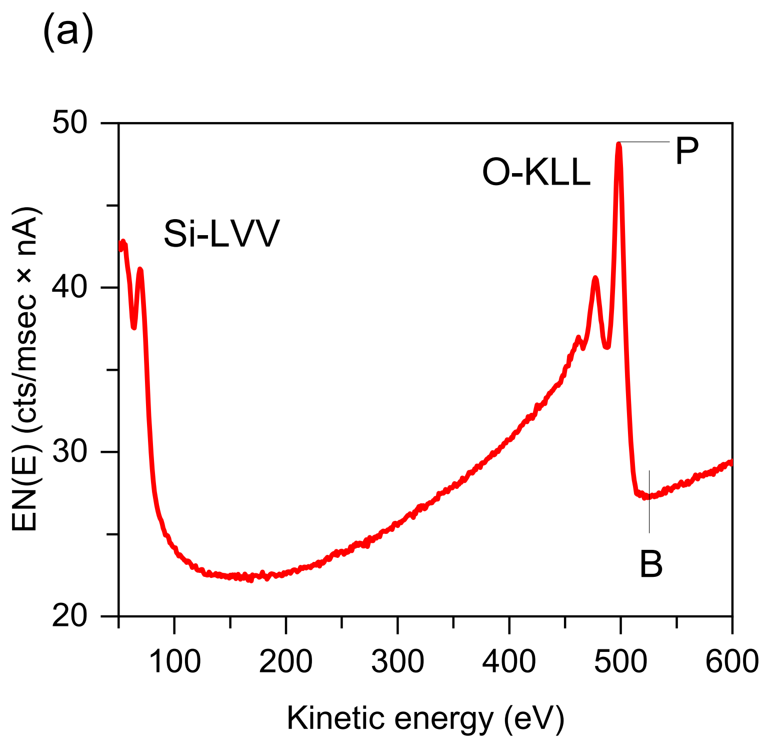

Figure S5. Auger electron spectra acquired on the reference materials: a) SiO_2 substrate, b) MoS_2 reference bulk crystal, with the marked positions for peak (P) and background (B) in O-KLL, S-LVV and Mo-MNN transitions used in the quantitative operations. The intensities used throughout the paper are defined as P−B for each of the three elements.

The starting point that facilitates a reliable Auger analysis is reference Auger spectra acquired for the investigated material, a stoichiometric SiO_2 and MoS_2 in our case. The reference Auger spectra enable the determination of reference intensities for respective Auger transitions in the given material. Typically, the reference spectra are derived from amorphous bulk solids. For this purpose, we utilise the SiO_2 substrate on which the samples were grown and a bulk MoS_2 standard purchased from hq Graphene. Prior to the measurement, the bare oxide substrate was gently cleaned with 500 eV Ar ions and then measured to collect the reference O-KLL Auger spectrum. The bulk MoS_2 standard was exfoliated by a scotch tape, and the clean exposed face of the crystal was then used as the reference for S-LVV and Mo-MNN transitions.

Figure S5(a) shows the reference for SiO_2 with Si-LVV and O-KLL transitions at 75 eV and 503 eV; **figure S5(b)** shows the MoS_2 reference with S-LVV (148 eV) and Mo-MNN (184 and 221 eV) transitions. Both spectra are normalised to (msec × nA) and the reference values for O, S, Mo intensities are determined by calculating the difference between the peak and background counts (P−B) for the respective elements yielding the following values: I_O^{∞}= 21.3 cts/msec×nA, I_S^{∞}= 33.2 cts/msec×nA, I_{Mo}^{∞}= 3.4 cts/msec×nA.

In quantitative operations, I_O^{∞} can be used directly to estimate of the thickness of the 2D layers deposited on the oxide substrates. The thickness is determined by the exponential attenuation of the I_O^{∞} signal coming from the substrate covered by the overlayer.
However, I_S^{∞} and I_{Mo}^{∞} cannot be used directly for the stoichiometric assessment of S and Mo species in atomically thin layers. This is because the reference intensities are derived from the bulk MoS_2, and the values must be corrected. The corrections are necessary for:

a/ the different values in electron backscattering in MoS_2 bulk vs. SiO_2 support,
b/ the different monolayer vs. bulk contributions in S-LVV and Mo-MNN signals.

The backscattering correction factor (BCF), which accounts for extra Auger excitation due to different backscattering of the primary beam in different materials, can be

estimated from the NIST SRD 154 database [31]. As demonstrated in **table S2**, the BCF ratio for MoS_2 bulk vs. SiO_2 support in S-LVV and Mo-MNN transitions has minimal impact on the I_{Mo}^{∞} / I_{S}^{∞} and therefore may be neglected.

Table S2. Backscattering correction factors from NIST SRD154 for incidence angle 30°, emission angle 30° and 10 keV excitation.

substrate	BCF_S	BCF_{Mo}	BCF_{Mo}/BCF_S
**MoS_2 on SiO_2**	1.474	1.686	1.144
**Reference bulk MoS_2**	1.593	1.843	1.157

In contrast, the contributions of the individual layers in MoS_2 bulk to I_{S}^{∞} and I_{Mo}^{∞} are different for S-LVV and Mo-MNN Auger electrons due to slightly different effective attenuation lengths (EAL). Consequently, the intensity ratio of the two will differ in bulks vs. monolayers; the attenuation lengths λ_{S}^{MS} and λ_{Mo}^{MS} for S-LVV and Mo-MNN electrons travelling through MoS_2 are 13% higher in the case of Mo (**figure S4**). According to the exponential approximation [26], the I_{S}^{∞} and I_{Mo}^{∞} values corrected for the monolayer are:

$$I_{S}^{1} = I_{S}^{\infty}\{1 - \exp\left[-d_0/\left(\lambda_{S}^{MS}\, cos\theta\right)\right]\} \qquad \text{S1(a)}$$

$$I_{Mo}^{1} = I_{Mo}^{\infty}\{1 - \exp\left[-d_0/\left(\lambda_{Mo}^{MS}\, cos\theta\right)\right]\} \qquad \text{S1(b)}$$

where d_0 = 0.62 nm is the thickness in MoS_2 monolayer and θ is the emission angle in the Auger experiment (30° in our case). The **equations S1(a)** and **S1(b)** yield the correct values for the monolayer:

I_{S}^{1} = 27.2 cts/msec×nA and I_{Mo}^{1} = 2.65 cts/msec×nA.

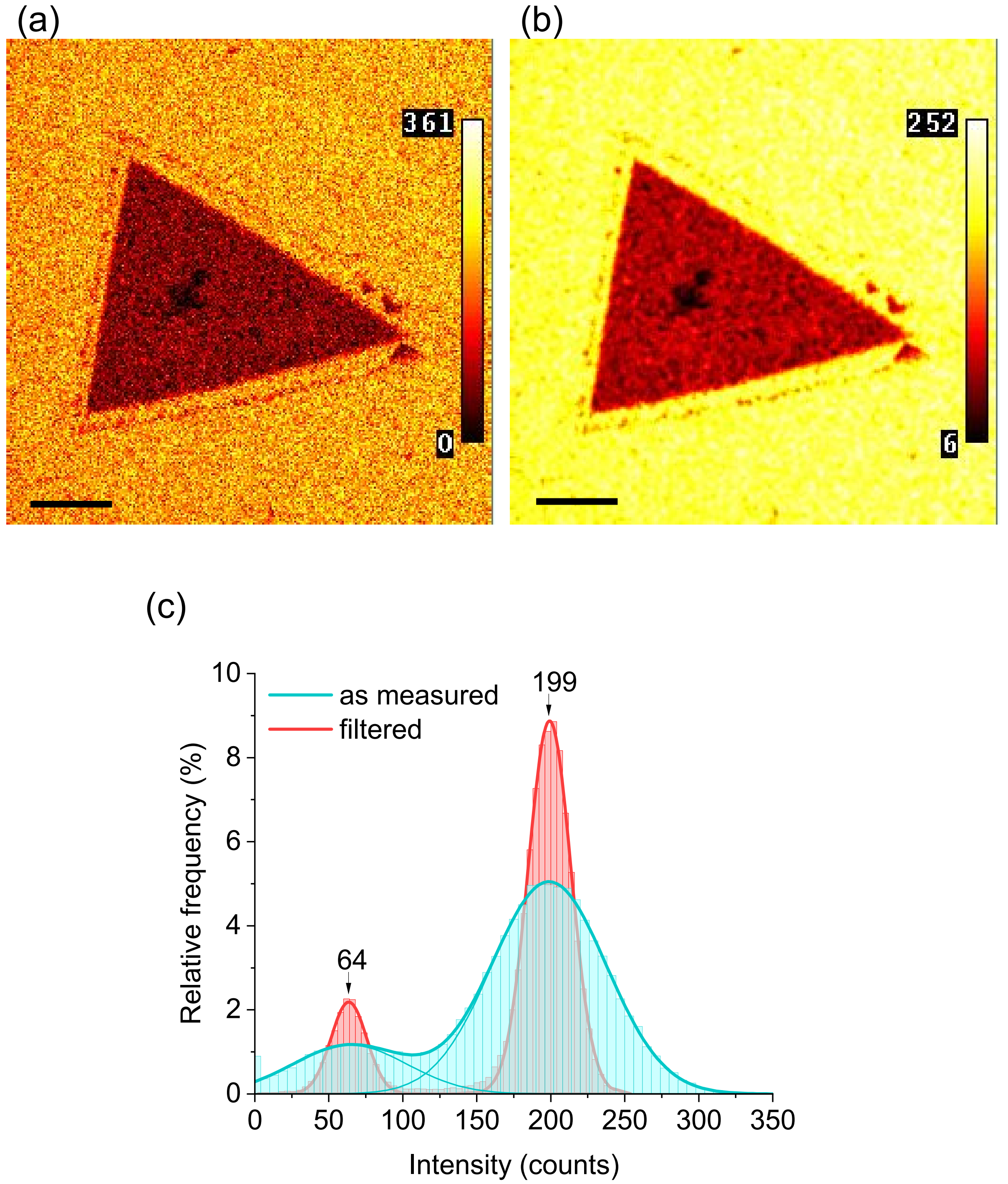

Figure S6. Oxygen Auger maps from a V-doped MoS_2 grain. (a) as-measured map, (b) same map modified by linear box filter. (c) histograms for the as-measured and the filtered map demonstrating the unchanged mean values but altered standard deviations due to the filtering procedure. The matrix for the linear box filter used in the smoothing was

$\frac{1}{9}\begin{bmatrix}1 & 1 & 1\\1 & 1 & 1\\1 & 1 & 1\end{bmatrix}$. The scalebar is 20 μm.

(a) (b) (c)

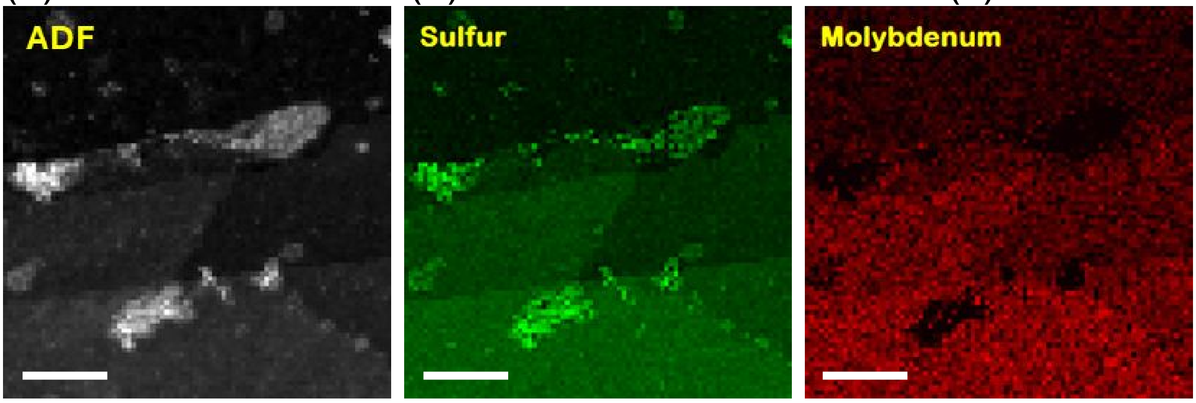

Figure S7. STEM investigations of MS layers. (a) annular dark field image. (b) Sulphur EELS map. (c) Molybdenum EELS map. The images show S contamination on the surface at places with an excess of S signals and lacking Mo signals. The scalebar is 100 nm.

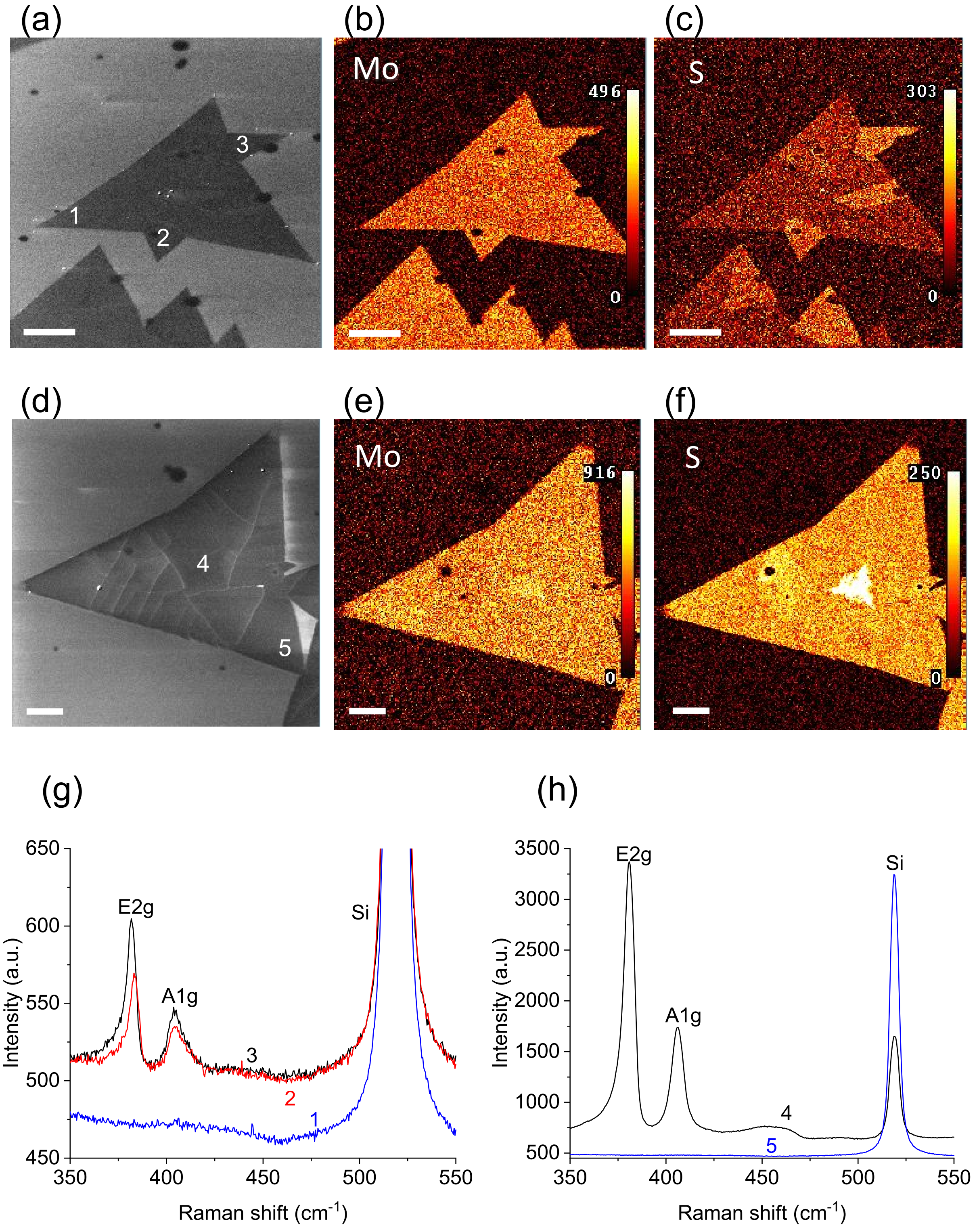

Figure S8. Creation of S-deficient and disordered 2D MoS_2 flakes in Ar/H_2 downstream plasma. (a), (d) SEM micrographs of two different as-grown grains. (b), (e) corresponding Auger maps for Mo after 30 sec of plasma treatment. (c), (f) corresponding Auger maps for S after 30 sec of plasma treatment, (g) and (h) Raman spectra on the plasma-treated grains at selected areas with different layering. The scalebar is 20 μm.
